# A Potential Novel Molecular Interaction in Bronchiolitis Obliterans Syndrome in Lung Transplantation Patients: The Role of SERPINA3 and Osteoprotegerin

**DOI:** 10.1096/fj.202503694R

**Published:** 2026-04-20

**Authors:** Yanzhe Liu, Eline A. van der Ploeg, Theo Borghuis, R. Ian Menz, Wim Timens, Judith M. Vonk, Barbro N. Melgert, C. Tji Gan, Janette K. Burgess

**Affiliations:** ^1^ Department of Pathology and Medical Biology, University Medical Center Groningen, University of Groningen Groningen the Netherlands; ^2^ Groningen Research Institute for Asthma and COPD (GRIAC), University Medical Center Groningen, University of Groningen Groningen the Netherlands; ^3^ Department of Pulmonary Medicine, University Medical Center Groningen, University of Groningen Groningen the Netherlands; ^4^ Faculty of Science, School of Life Sciences, University of Technology Sydney Sydney New South Wales Australia; ^5^ Department of Epidemiology, University Medical Centre Groningen, University of Groningen Groningen the Netherlands; ^6^ Department of Molecular Pharmacology, Groningen Research Institute for Pharmacy, University of Groningen Groningen the Netherlands

**Keywords:** airway obstruction, airway remodeling, bronchiolitis obliterans syndrome, chronic lung allograft dysfunction, fibrosis, lung transplantation, TNFRSF11b

## Abstract

Chronic lung allograft dysfunction significantly limits survival after lung transplantation. The obstructive phenotype bronchiolitis obliterans syndrome (BOS) is characterized by the abnormal activation of airways epithelium, fibrotic changes with excessive extracellular matrix deposition, and airway obliteration. Mast cells, through mediators such as tryptase and chymase, play a role in lung fibrosis. The proteins osteoprotegerin (OPG) and SERPIN family member A 3 (SERPINA3) have been associated with lung fibrosis progression. Tryptase‐mast cells can produce OPG, while chymase interacts with SERPINA3. This study aimed to investigate if and how SERPINA3, OPG, and tryptase/chymase‐positive mast cells are related to fibrotic airway obliteration and potentially show an association with BOS severity. Serum SERPINA3 levels in BOS and non‐BOS were examined using ELISA. In BOS lung tissue, non‐cartilaginous airways were classified into normal, partially obstructed, and completely obstructed airways. Immunohistochemistry detected SERPINA3, OPG, chymase, and tryptase. Colocalization of and interactions between SERPINA3, OPG, and tryptase were assessed by immunofluorescence, proximity ligation assay, and AlphaFold modeling. SERPINA3 serum levels in BOS patients were higher compared to non‐BOS patients. A low percentage of SERPINA3 and OPG was detected in partially and completely obstructed airways. Cells positive for OPG and SERPINA3 colocalized with tryptase‐mast cells in airways. OPG colocalized with SERPINA3, and they positively correlated in partially obstructed airways. In completely obstructed airways, OPG, SERPINA3, and tryptase all positively correlated with each other. These findings suggest mast cells express SERPINA3 and OPG, and these proteins potentially form a complex in lung tissue, possibly contributing to airway remodeling in BOS.

## Graphical abstract

Serum SERPINA3 levels were higher in BOS compared to non‐BOS patients. OPG and SERPINA3 positive cells colocalized with tryptase positive mast cells in airways from BOS lung tissue. OPG and SERPINA3 staining positively correlated in partially and completely obstructed airways. SERPINA3 and OPG may form a complex in lung tissue potentially contributing to airway remodeling in BOS.

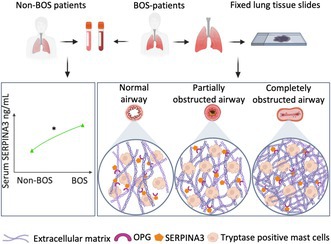

## 1 Introduction

Chronic lung allograft dysfunction (CLAD) limits survival after lung transplantation [1]. Bronchiolitis obliterans syndrome (BOS) is the most common form of CLAD, affecting 50% of patients 5 years after lung transplantation [2]. BOS is a clinical diagnosis based on persistent lung function decline of at least 20% of forced expiratory volume in 1 s (FEV1) compared to baseline after lung transplantation [3]. Bronchiolitis obliterans is defined as a histopathological hallmark of BOS. It is characterized by an abnormal repair process in predominantly non‐cartilaginous, smaller airways, which includes initial epithelial inflammation with subsequent fibrosis formation. Excessive deposition of extracellular matrix [4] in the submucosa of airways then ultimately contributes to complete obstruction with the absence of any remaining epithelium in smaller airways [5]. Such fibrotic abnormalities result in partial or complete obliteration of the airway lumen, leading to a decline of lung function and symptoms of breathlessness [6]. However, the mechanisms underlying bronchiolitis obliterans remain poorly understood.

Mast cells play a critical role in abnormal repair processes by releasing mediators that drive extracellular matrix remodelling [7]. Tryptase, which is released from mast cells, is also recognized as a marker to identify mast cells [8]. Chymase, another mediator secreted from a subset of mast cells, plays a crucial role in orchestrating extracellular matrix degradation by regulating the activation of matrix metalloproteases to maintain tissue homeostasis [9]. Chymase interacts with SERPIN family member A3 (SERPINA3), also known as alpha‐1‐antichymotrypsin, to form an irreversible chymase‐SERPINA3 complex which inhibits chymase activity [10]. We recently reported that SERPINA3 was higher in serum from patients with BOS after lung transplantation compared to non‐BOS patients [11]. SERPINA3 is an acute phase protein that is secreted by the liver in response to inflammation [12] and has been linked to fibrotic responses in various tissues. In a model of bleomycin‐induced lung fibrosis, high levels of SERPINA3 were found, and silencing an isoform of SERPINA3 significantly reduced expression of collagen type I and fibronectin [13]. Furthermore, *SERPINA3* gene expression positively correlated with expression of collagen type II A1 mRNA in cartilage, and its silencing downregulated expression of genes related to assembly of collagen fibrils in mesenchymal stem cells [14]. Whether disruption of the relationship between SERPINA3 and chymase is an important driver of the fibrotic extracellular matrix changes observed in BOS lung disease has yet to be examined.

Osteoprotegerin (OPG), a member of the tumor necrosis factor (TNF) receptor superfamily, is a glycoprotein that plays a key role in regulating homeostasis of bone extracellular matrix [15]. Beyond its traditional function in bone, OPG has increasingly been implicated in various fibrotic conditions. Elevated serum OPG levels, along with its significant presence in lung tissues in patients with idiopathic pulmonary fibrosis (IPF), have been observed. Elevated serum OPG was also associated with decreased lung function and progression of IPF [16]. Moreover, a recent study has identified OPG as a marker of early fibrosis and an indicator of responsiveness to anti‐fibrotic therapy [17]. Importantly, elevated levels of serum OPG were strongly associated with graft failure and related to all‐cause mortality in renal transplantation recipients [18, 19]. Of interest, tryptase‐positive mast cells are capable of secreting OPG [20] to regulate bone turnover.

Building on these findings, this study aimed to investigate if SERPINA3, OPG, and chymase/tryptase‐positive mast cells were related to airway obliteration, potentially contributing to BOS severity. We hypothesized that enhanced OPG production by tryptase‐positive mast cells interacts with the increased SERPINA3 to contribute to the airway obstruction of BOS. To investigate this hypothesis, we identified multiple non‐cartilaginous airways with varying degrees of submucosal fibrosis in lung tissue from patients with BOS after lung transplantation to detect the presence of SERPINA3, OPG, and chymase/tryptase‐positive mast cells in relation to fibrosis progression in BOS.

The manuscript has been uploaded to *BioRXiv* [21].

## 2 Material and Methods

### 2.1 Ethics Information

For serum analysis, lung transplant (LTx) patients who underwent bilateral lung transplantation between 2004 and 2017 in the University Medical Center Groningen were screened. BOS patients who progressed to stage three according to the International Society of Heart and Lung Transplantation Guidelines were selected if longitudinal serum samples were available. BOS patients were matched to non‐BOS patients for sex, age at LTx, diagnosis necessitating LTx, immunosuppression, and storage time of the samples (Table 1). Explants from LTx patients who received a re‐transplantation due to BOS or deceased with end stage BOS were collected for tissue analysis. All patients received immunosuppression according to protocol (Table 2). Patients provided written informed consent for use of material. The study was approved by the medical ethics committee of the University Medical Center Groningen (METc 2014/077, METc 2021/610, research register number: 202000737), adheres to the UMCG Biobank Regulation, and was conducted in accordance with the WMA Declaration of Helsinki and Declaration of Istanbul. Patients were enrolled in the ongoing, prospective TransplantLines Biobank and Cohort Study (ClinicalTrials.gov identifier: NCT03272841), in which, since June 2015, all (potential) solid organ transplantation patients and (potential) living organ donors (aged ≥ 18 years) at the University Medical Center Groningen (UMCG, The Netherlands) have been invited to participate.

**TABLE 1:** Patient characteristics of BOS and non‐BOS patients for serum analysis.

	BOS (*n* = 19)	Non‐BOS (*n* = 19)	*p*
Patient sex, female (%)	12 (63.2%)	12 (63.2%)	1.0
Age at LTx (y)	55 [44–66]	55 [43–66]	0.6
Underlying disease			0.7
COPD	9^a^	11^a^	
CF	2	2	
AATD	5	3	
Scleroderma	1	1	
Other	2^b^	2^b^	
Donor sex, female (%)	12 (63.2%)	11 (57.9%)	1.0
Donor age	52 ± 10	46 ± 12	0.1
Donor pack years	2.5 [0–12.5]	0 [0–15]	0.8
Immunosuppression after LTx			1.0
Tacrolimus/azathioprin/prednisolon	5 (26.3%)	5 (26.3%)	
Tacrolimus/mmf/prednisolon	14 (73.7%)	14 (73.7%)	
Patients with documented diagnosis of viral infection after LTx	10 (52.6%)	3 (15.8%)	0.08
Time to diagnosis of BOS (y)	2.8 [1.9–5.8]	NA	
Pulmonary function test—in L (*n*)^c^			
FEV1 BOS stage 1	1.97 ± 0.64 (18)	2.78 ± 0.39 (18)	< 0.001
FVC BOS stage 1	3.18 ± 0.81 (18)	3.81 ± 0.89 (18)	0.1
FEV1 BOS stage 2	1.59 ± 0.53 (18)	2.76 ± 0.47 (8)	< 0.001
FVC BOS stage 2	3.03 ± 0.88 (18)	3.57 ± 0.93 (8)	0.17
FEV1 BOS stage 3	1.21 ± 0.39 (19)	2.70 ± 0.46 (16)	< 0.001
FVC BOS stage 3	2.58 ± 0.81 (19)	3.74 ± 0.89 (16)	< 0.001
FEV1% predicted BOS stage 1	71.50 ± 15.06 (18)	95.50 ± 24.14 (18)	< 0.001
FVC% predicted BOS stage 1	90.5 ± 16.30 (18)	106.00 ± 18.59 (18)	0.006
FEV1% predicted BOS stage 2	53.33 ± 13.08 (18)	105.25 ± 20.21 (8)	< 0.001
FVC% predicted BOS stage 2	84.28 ± 15.49 (18)	110.88 ± 15.35 (8)	< 0.001
FEV1% predicted BOS stage 3	40.0 ± 8.98 (19)	98.00 ± 19.34 (16)	< 0.001
FVC% predicted BOS stage 3	70.00 ± 14.56 (19)	107.00 ± 12.55 (16)	< 0.001

Abbreviations: AATD, alpha‐1 anti‐trypsin deficiency; BOS, bronchiolitis obliterans syndrome; CF, cystic fibrosis; COPD, chronic obstructive pulmonary disease; FEV1% predicted, forced expiratory volume as a percentage of the predicted value; FEV1, forced expiratory volume in 1 s; FVC% predicted, forced vital capacity as a percentage of the predicted value; FVC, forced vital capacity; LTx, lung transplantation; m, months; MMF, mycophenolic acid; *n*, number; y, years.

^a^ 2 BOS patients with alpha‐1 antitrypsin deficiency were matched to non‐BOS COPD controls.

^b^ One BOS patient with histiocytosis X matched with a non‐BOS patient with pulmonary fibrosis. One BOS patient with pulmonary fibrosis after infection matched with non‐BOS patient with selective IgG2 deficiency and bronchiectasis.

^c^ Non‐BOS patients (*n* = 19) were matched to BOS patients (*n* = 19) for time after transplantation regarding pulmonary function tests performed.

**TABLE 2:** Patient characteristics of BOS patients (*n* = 6) for tissue analysis.

	BOS (*n* = 6)
Patient sex, female (%)	1 (16.7%)
Age at first LTx (y)	35 [20–48]
Time until re‐LTX or death (y)	6.1 [4.0–10.7]^a^
Underlying disease	
CF	2 (33.3%)
AATD	4 (66.7%)
Donor age	45 [29–50]
Donor pack years	0 [0–3]
Pulmonary function test—L^b^	
FEV1 BOS stage 1	2.4 [1.9–2.8]
FVC BOS stage 1	4.1 [3.6–4.8]
FEV1 BOS stage 3	1.4 [1.1–1.9]
FVC BOS stage 3	3.1 [2.9–3.7]
FEV1% predicted BOS stage 1	68.0 [51.8–72.3]
FVC% predicted BOS stage 1	82.5 [78.0–90.0]
FEV1% predicted BOS stage 3	42.5 [33.0–46.0]
FVC% predicted BOS stage 3	69.5 [67.3–88.3]

Abbreviations: AATD, alpha‐1 anti‐trypsin deficiency; BOS, bronchiolitis obliterans syndrome; CF, cystic fibrosis; FEV1, forced expiratory volume in 1 s; FVC, forced vital capacity; L, liters; LTx, lung transplantation; *n*, number; y, years.

^a^ 5 BOS patients underwent re‐transplantation because of BOS; one patient was included after obduction.

^b^ Pulmonary function testing of 4 patients included. Missing values due to first transplantation and follow up in other centers.

### 2.2 Patient Information

The patient cohort used in this study for the detection of SERPINA3 in serum was the same as that used in our previous proteomic study [22]. Briefly, non‐BOS patients (*n* = 19) were matched to BOS patients (*n* = 19). Serum samples were identified that had been collected at 12, 6, and 3 months before BOS onset, and subsequently at BOS stage 2 and BOS stage 3. We also matched the time points of collection of samples for the non‐BOS patients to BOS patients. The information relating to this cohort for the serum SERPINA3 analysis is in Table 1, and the cohort used for detection of SERPINA3, tryptase, OPG, and chymase in lung tissue is in Table 2.

### 2.3 Soluble SERPINA3 Detection

ELISA was used to assess the level of SERPINA3 in serum, Human alpha 1‐Antichymotrypsin ELISA Kit (ab157706, Abcam, Cambridge, United Kingdom), using a sample dilution of 1:5000, optimized according to the manufacturer's instructions.

### 2.4 Lung Tissue Staining

Structures within lung tissue were identified using hematoxylin and eosin (H&E), Verhoeff's, and Martius Scarlet Blue (MSB).

### 2.5 Hematoxylin & Eosin (HE) Staining

Three μm sections of paraffin‐embedded lung tissues from patients with BOS were deparaffinized in xylol for two times 10 min. Subsequently, the sections were washed two times with 100% ethanol, with 96% ethanol, with 70% and with demi water each for 10 s. Then hematoxylin was stained in the slides for 10 min. After washing the slides with tap water and incubating the slide in tap water for 10 min, the slides were stained with eosin for 2 min. Next, the sections were dehydrated with 96% ethanol, and with 100% each for 10 s. Slides were air‐dried for 10 min before mounting the slides with mounting medium (SP15100, Thermo Fisher Scientific, United States). Finally, the slides were scanned at 40× magnification using the slide scanner Hamamatsu Nanozoomer (Hamamatsu Photonic K.K., Japan).

### 2.6 Verhoeff Staining

Three μm sections of paraffin‐embedded lung tissues from patients with BOS were deparaffinized in xylol twice for 10 min. The sections were then incubated in demi water to remove residual xylene. Subsequently, slides were incubated in Verhoeff working solution for 15 min, followed by a brief rinse in demi water. Next, slides were incubated with 1% ferric chloride for 1 min, followed by washing slides with demi water three times. Then, slides were incubated in van Gieson's stain solution for 2 min and washed with demi water after staining. Dehydrated slides in 100% alcohol, air‐dried, and mounted with mounting media (Thermo Fisher Scientific, United States). Slides were scanned at 40× magnification using the slide scanner Hamamatsu Nanozoomer (Hamamatsu Photonic K.K., Japan); reagents used in Verhoef staining were summarized in Table 3.

**TABLE 3:** Reagen using in Verhoeff staining.

Reagent	Composition
Verhoeff Stock Solution (A)	5 g hematoxylin in 100 mL 100% ethanol
Verhoeff Stock Solution (B)	5 g ferric chloride in 50 mL demi water
Verhoeff Stock Solution (C)	1 g potassium iodide in 2.5 mL demi water, then add 0.5 g iodine.
Verhoeff working solution	25 mL Solution A + 10 mL Solution B + 10 mL Solution C
Acid fuchsin	0.1 g acid fuchsin in 10 mL distilled water
Van Gieson's solution	6 mL saturated picric acid +1 mL 1% acid fuchsin
Ferric chloride	2 g in 100 mL demi water

### 2.7 Immunohistochemical Staining

SERPINA3, tryptase, chymase and OPG were detected in BOS lung tissue using immunohistochemistry. Three μm sections of paraffin‐embedded lung tissues from patients with BOS were deparaffinized and antigens were retrieved through incubating in 10 mM citrate buffer at pH 6.0 at 100°C for 15 min. Endogenous peroxidases were blocked in a solution of PBS containing 0.3% hydrogen peroxide (H_2_O_2_) (Merck KGaA, Darmstadt, Germany) at room temperature for 30 min. After washing three times in 1× Phosphate Buffered Saline (PBS), lung tissue sections were blocked with 4% bovine serum albumin (BSA) in PBS at room temperature for 30 min. Next the sections were incubated with primary antibodies overnight at 4°C, then incubated with secondary antibodies for 45 min at room temperature (Table 4), before being washed with PBS three times and subsequently with demi water three times. Finally, the staining was visualized by incubating the slides for 10 min with Nova Red staining solution (SK‐4800, BRUNSCHWIG CHEMIE, Amsterdam, Netherlands). All sections were counterstained using hematoxylin, before being dehydrated and mounted with histological mounting media (Thermo Fisher Scientific, United States). Slides were scanned at 40× magnification using slide scanner Hamamatsu Nanozoomer (Hamamatsu Photonic K.K., Japan). The specificity of the antibody stains were checked using an isotype control (Figure S1).

**TABLE 4:** Antibodies used in immunohistochemical staining.

Primary antibody	Primary antibody source	Primary antibody dilution	Secondary antibody	Secondary antibody source	Secondary antibody dilution
Anti‐AACT	ab180492, Abcam	1:1000	Goat anti‐rabbit immunoglobulin‐HRP	Dako P0488, Dako, Amsterdam, NL	1:100
Anti‐mast cell Tryptase	ab2378, Abcam	1:100 000	Rabbit anti‐mouse immunoglobulin‐HRP	Dako P0260	1:100
Anti‐chymase	JB74‐320, Novus Biologicals, Abingdon, United Kingdom	1:16 000	Goat anti‐rabbit immunoglobulin‐HRP	Dako P0488	1:100
Anti‐osteoprotegerin	ab183910, Abcam	1:300	Goat anti‐rabbit immunoglobulin‐HRP	Dako P0488	1:100
Rabbit IgG isotype control X0903 Antibody	X0903, DAKO A/S, Denmark	1:12.500	Goat anti‐rabbit immunoglobulin‐HRP	Dako X0903	1:100

Abbreviation: AACT, alpha‐1‐antichymotrypsin.

### 2.8 Immunofluorescent Staining

To investigate (co)localization of above proteins in BOS lung tissue we used immunofluorescence. For simultaneous detection of SERPINA3, OPG and Tryptase, sections of formalin‐fixed paraffin embedded lung tissue were sequentially stained for each protein with tyramide signal amplification. This method allows the detection of multiple primary antibodies without species cross‐reactivity and is based on an antibody stripping protocol which removes primary antibodies and secondary antibodies but not the visualization signal. Sections of formalin‐fixed paraffin embedded lung tissue were deparaffinized, incubated with citrate buffer (10 mM sodium citrate, pH 6) for 15 min at 100°C for antigen retrieval, and cooled down for 30 min at room temperature. The slides were washed with Tris Buffered Saline (TBS), and endogenous peroxidase was blocked by incubating the slides in TBS containing 0.3% hydrogen peroxide for 30 min at room temperature. The slides were incubated with rabbit anti‐SERPINA3 (Abcam, ab180492, 1:500) in TBS with 1% BSA for 1 h at room temperature followed by goat anti‐rabbit horseradish peroxidase‐conjugated secondary antibody (DAKO, P0448, 1:100). Visualization was performed using tyramide signal amplification process with Opal 570 Reagent Pack (AKOYA Biosciences, FP1488001KT); slides were incubated in Opal 570 Reagent Pack diluted 1:200 in 0.1 M Borate buffer containing 0.003% H_2_O_2_ for 10 min at room temperature. Next, slides were incubated again in citrate buffer (10 mM sodium citrate, pH 6) for 15 min at 100°C to remove all antibodies and cooled down for 30 min at room temperature. The slides were then incubated with mouse anti‐Tryptase (Abcam, ab2378, 1:10 000) and rabbit anti‐OPG (Abcam, ab183910, 1:300) in TBS with 1% BSA for 90 min at room temperature followed by goat anti‐rabbit horseradish peroxidase‐conjugated secondary antibody (DAKO, P0448, 1:100) and donkey anti‐mouse Alexa647‐conjugated secondary antibody (Thermo, A‐31571, 1:500). Visualization for OPG was performed using tyramide signal amplification process with Opal 520 Reagent Pack (AKOYA Biosciences, FP1487001KT); slides were incubated in Opal 520 Reagent Pack diluted 1:200 in 0.1 M Borate buffer containing 0.003% H_2_O_2_ for 10 min at room temperature. Negative staining controls were performed by omitting the primary antibody incubations and by staining for only 1 protein but including all secondary steps for the other proteins. Nuclei were visualized with DAPI, and slides were mounted in citifluor (Thermo, 17970‐025). Fluorescent images were acquired using a Zeiss LSM780 confocal scanning microscope (Carl Zeiss, Oberkochen, Germany) and an Olympus VS200 fluorescent slide scanner (Olympus Corporation, Tokyo, Japan).

### 2.9 Proximity Ligation Assay

Protein–protein interactions between our proteins were studied using the proximity ligation assay with the Nave Bright‐HRP kit (NB.MR.HRP.100, Navinci, Uppsala, Sweden). The kit enables chromogenic visualization of protein–protein interactions. Sections of formalin‐fixed paraffin embedded lung tissue were deparaffinized and subsequently incubated with citrate buffer (10 mM sodium citrate, pH 6) for 15 min at 100°C for antigen retrieval. The slides were cooled down for 30 min at room temperature and washed with TBS. Endogenous peroxidase was blocked by incubating the slides in TBS containing 0.3% hydrogen peroxide for 30 min at room temperature. The slides were washed with TBS and then incubated overnight with four different combinations of primary antibodies: mouse anti‐SERPINA3 (Proteintech, 66078‐1—Ig, 1:100) + rabbit anti‐OPG (Abcam, ab183910, 1:300), mouse anti‐tryptase (Abcam, ab2378, 1:10 000) + rabbit anti‐OPG (Abcam, ab183910, 1:300) and mouse anti‐tryptase (Abcam, ab2378, 1:10 000) + rabbit anti‐SERPINA3 (Abcam, ab180492, 1:1000). Also, incubations with only one primary antibody were taken along to assess background staining. All antibodies were diluted in TBS containing 2% BSA. After washing with TBS 0.1% TWEEN 20 (P1379, Sigma‐Aldrich, Amsterdam, Netherlands), the slides were incubated with anti‐mouse Navenibody and anti‐rabbit Navenibody in Navenibody diluent at 37°C for 1 h, followed by enzymatic ligation, rolling PCR amplification, HRP incubation and substrate development according to the manufacturer's instructions (Navinci, NB.MR.HRP.100). Nuclei were visualized with Mayer's hematoxylin (Sigma Aldrich). Slides were scanned using a Hamamatsu NanoZoomer 2.0 HT digital scanner (Hamamatsu Photonics).

### 2.10 Protein Interaction Modeling

To predict potential biomolecular interactions between SERPINA3, tryptase, chymase, and OPG we used AlphaFold Server [23] (https://alphafoldserver.com). The strength of the predicted protein–protein interactions was assessed using PPCheck [24] (https://caps.ncbs.res.in/ppcheck/). Open Babel [25] version 3.1.1 hosted on cheminfo.org was used to convert structure file formats. Molecular graphics images were produced using UCSF Chimera [26] version 1.17. Prediction of antigenic peptides was performed using the Universidad Complutense Madrid, Immunomedicine Group tool (http://imed.med.ucm.es/Tools/antigenic.pl) using the method of Kolaskar and Tongaonkar [27].

### 2.11 Airway Categorization, Identification and Annotation

**FIGURE 1:**
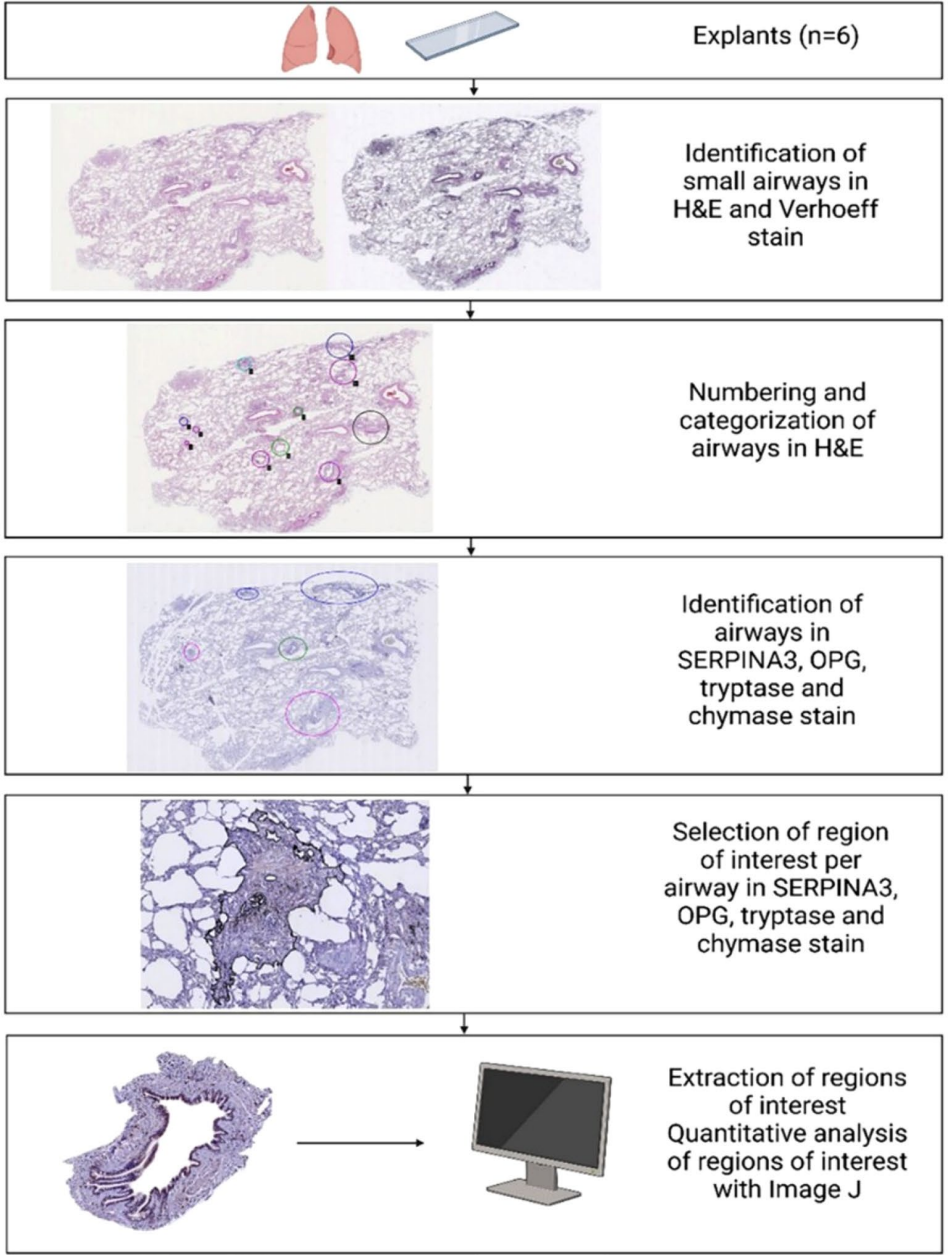
Flow diagram of identification of airway, airway categorization and selection of region of interest. Explant tissue (*n* = 6) was stained with hematoxylin and eosin (H&E), as well as Verhoef stain. Airways were identified in H&E and Verhoeff stain. Airways were numbered and categorized into different airway categories (normal, partially obstructed, completely obstructed). Airways were also identified in SERPINA3, tryptase, chymase and OPG stained sections and categorized according to the classification code established in the H&E‐stained section. Region of interest defined as the outer border of the adventitia of the airway to the inner border of the airway lumen for completely, partially and normal airways, was identified and selected. Regions of interest were extracted and quantitative analysis was performed using Image J. H&E, hematoxylin and eosin; *N*, number; OPG, osteoprotegerin; SERPINA3, SERPINA family member A3.

**FIGURE 2:**
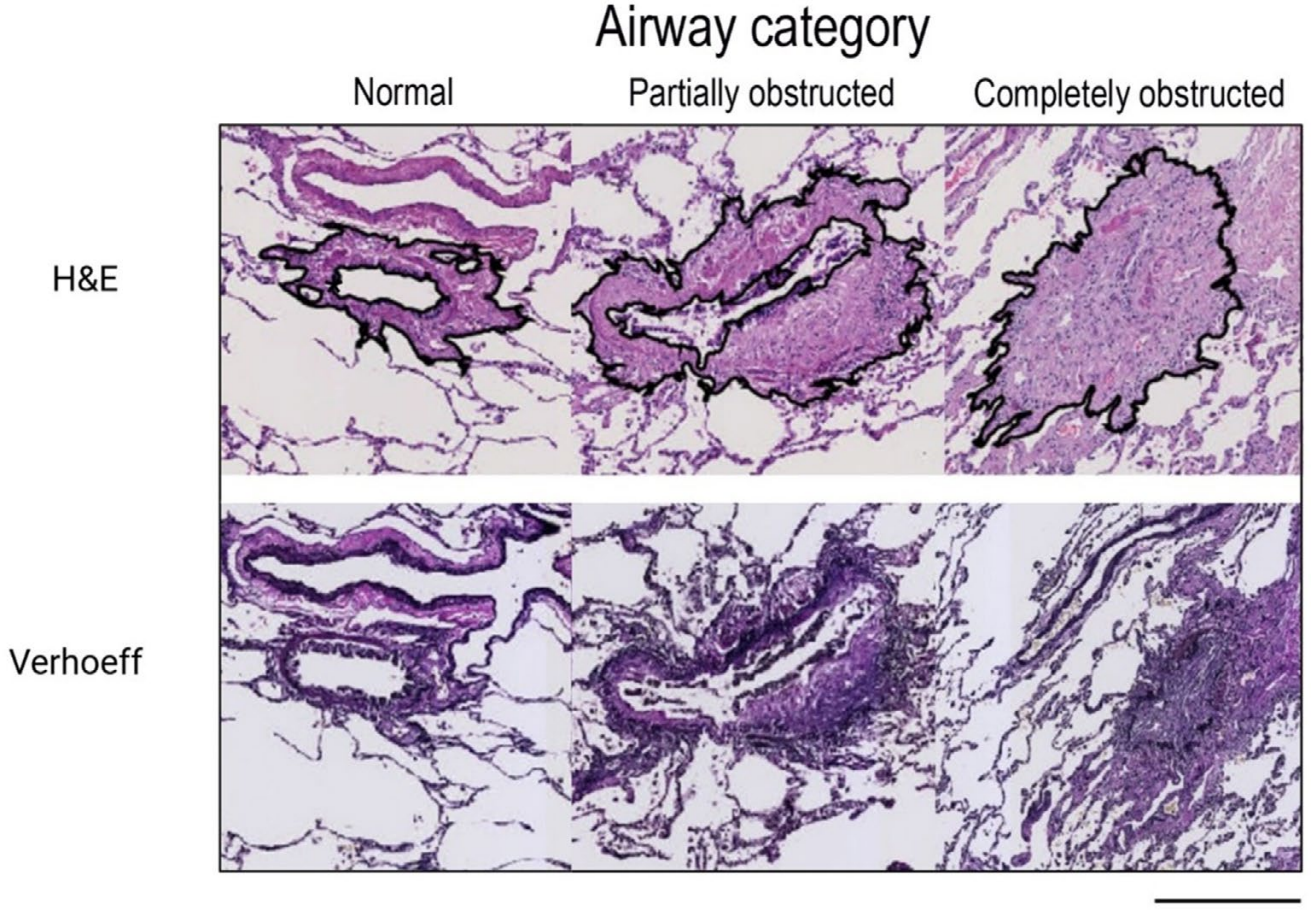
Airway categorization and identification of region of interest per airway category. Upper row shows airway categories in hematoxylin and eosin, bottom row shows airway categories in Verhoeff stain. Black line indicates region of interest, defined as the outer border of the adventitia of the airway to the inner border of the airway lumen for partially and normal airways. When airways were completely obstructed, the circumference of the outer border of the adventitia was identified as the boundary of the region of interest. Definition of airway categories: Normal; airway not affected by obliterative bronchiolitis, partially obstructed: Airway with fibrotic obliteration with a greater distance between the airway smooth muscle layer and the epithelial layer, with at least a partial recognizable lumen; completely obstructed: Airways that have a completely obstructed lumen. Magnification 40×, black scale bar 400 μm. HE, hematoxylin and eosin stain.

Slides from recipients with BOS were scanned and uploaded into Qupath [28], version 0.5.0. All airways in H&E and Verhoef‐stained lung tissue sections from patients with BOS (*n* = 6, 3–5 sections per patient) were identified by an experienced lung pathologist (WT), and all airways without presence of cartilage and submucosal glands were included. Airways that were excluded were the last part of the terminal bronchiole going into respiratory bronchioles (so where the airway smooth muscle cell layer was interrupted), because of the absence of ECM. For each airway, the airway region of interest was defined as the region between the outer border of the adventitia of the airway and the inner border of the airway lumen for normal and partially affected airways. When airways were completely obstructed, the circumference of the outer border of the adventitia was identified as the boundary of the region of interest (Figure 1). All airways were identified and categorized according to severity of obliteration (shown in Table 5). In this study, we focused on three airway categories, including normal airway, partially obstructed airway, and completely obstructed airway. Aberrations within the images that were excluded from the area of interest are described in Figure 2 and Table 6.

**TABLE 5:** Airway categorization and identification.

Categorization	Identification
Normal airway	Unaffected airways
Partially obstructed, non‐active airway	Airway with fibrotic obliteration with a greater distance between the airway smooth muscle layer and the epithelial layer than normal airway, with at least a partially recognizable lumen
Completely obstructed airway	Airways that have a completely obstructed lumen

**TABLE 6:** Features excluded from the regions of interest.

To be excluded area	Details
Lumen area in unaffected and partially obstructed airways	No cells present; not contributing to airway surface containing extracellular matrix
Arteriole in adventitia with diameter > 50 μm	Exclude if no clear arteriole wall; if wall is identifiable, exclude both lumen and wall
Larger vessels	Border determined by matrix change or muscle layer of vessel
Epithelial layer loose from basal membrane	If completely loose, exclude both epithelium and lumen; if still attached, include
Protrusions of airway with inflammatory infiltrate	Excluded due to the little extracellular matrix present in these areas and small protrusions without immune

The same airways were also identified in SERPINA3, tryptase, chymase, and OPG‐stained sections (3–5 sections per patient/per protein of interest) and categorized according to the classification code established in the H&E‐stained sections.

### 2.12 Image Analyses

All stained slides were scanned with a Nanozoomer (Hamamatsu Photonic K.K., Japan). NanoZoomer Digital Pathology Image (NDPI) files were first converted to TIF images in Aperio ImageScope (Leica) and subsequently processed in Adobe Photoshop 2024 (Adobe Inc. California, United States) to exclude artifact areas such as folded tissues and carbon‐based pigments. Next, Image J win 64 software [29] was used to quantify areas with positive staining. A summary of the sample size analyzed for each staining is shown in Table 7. For analyses of staining images, briefly, the NDPI files were firstly converted to TIF images in Aperio ImageScope (v12.3.3.5048, Leica) and subsequently processed in Adobe Photoshop 2024 (Adobe Inc. California, United States) to exclude artificial areas such as folded tissues and carbon‐based pigments. Next, Image J win 64 software was utilized to quantify the area with positive staining. All images were split into blue (hematoxylin‐image), and red (NovaRed‐image) pixels using color deconvolution plugin of Image J [30]. To calculate the total amount of tissue, images were converted to 8‐bit gray scale. Total number of pixels representing total tissue area versus positively stained tissue area were identified using the threshold feature of Image J. Percentage of positive area within the whole region was calculated using the formula below. Data analyses were performed with R software 4.4.0 (Boston, Massachusetts, USA).

$$\text{Area}\;\left(\%\right)=\frac{\text{Number of pixels positive for NovaRed}}{\text{Total number of pixels positive for tissue}}\times 100$$

**TABLE 7:** Summary of sample size analyzed for each staining.

Staining	Patient (*n*)	Block/patient (*n*)	Normal airway (*n*)	Partially obstructed airway (*n*)	Completely obstructed airway (*n*)
SERPINA3	6	2–5	9	46	33
Tryptase	6	2–5	8	56	29
Chymase	6	2–5	11	55	35
OPG	6	2–5	13	51	38

### 2.13 Statistical Analyses

Patient characteristics are described as mean ± standard deviation for normally distributed data, or median [interquartile range] for non‐normally distributed data. Data were tested for normality with a Shapiro Wilk test and visually assessed by histograms and P–P plots. If not normally distributed, data were Ln transformed to attain a normal distribution. If this did not yield a normal distribution, nonparametric tests were used when comparing two groups, i.e., Mann–Whitney *U* or student's *t*‐tests depending on normality of the data. Longitudinal data for serum SERPINA3 levels were examined with a linear mixed model using IBM SPSS Statistics 28.0.1.0 after Ln transformation of SERPINA3 levels for normalization, with intercept per patient as random effect. Differences in the percentages of positively stained area within sections of lung tissue between normal airways, partially and completely obstructed airways were assessed using linear mixed effects regression analysis in SPSS with two random effects on intercept, to correct for both per subject and paraffin block variability. For staining analysis natural log (ln) transformation was performed to achieve normality of the data. Correlations between SERPINA3, OPG and tryptase were assessed using linear regression with either Pearson or Spearman test depending on normality of the data. Correlation data are shown with 95% confidence bands of the best‐fit line. *p* < 0.05 was considered significant for all tests.

## 3 Results

### 3.1 Higher Serum SERPINA3 Level in Patients With BOS

**FIGURE 3:**
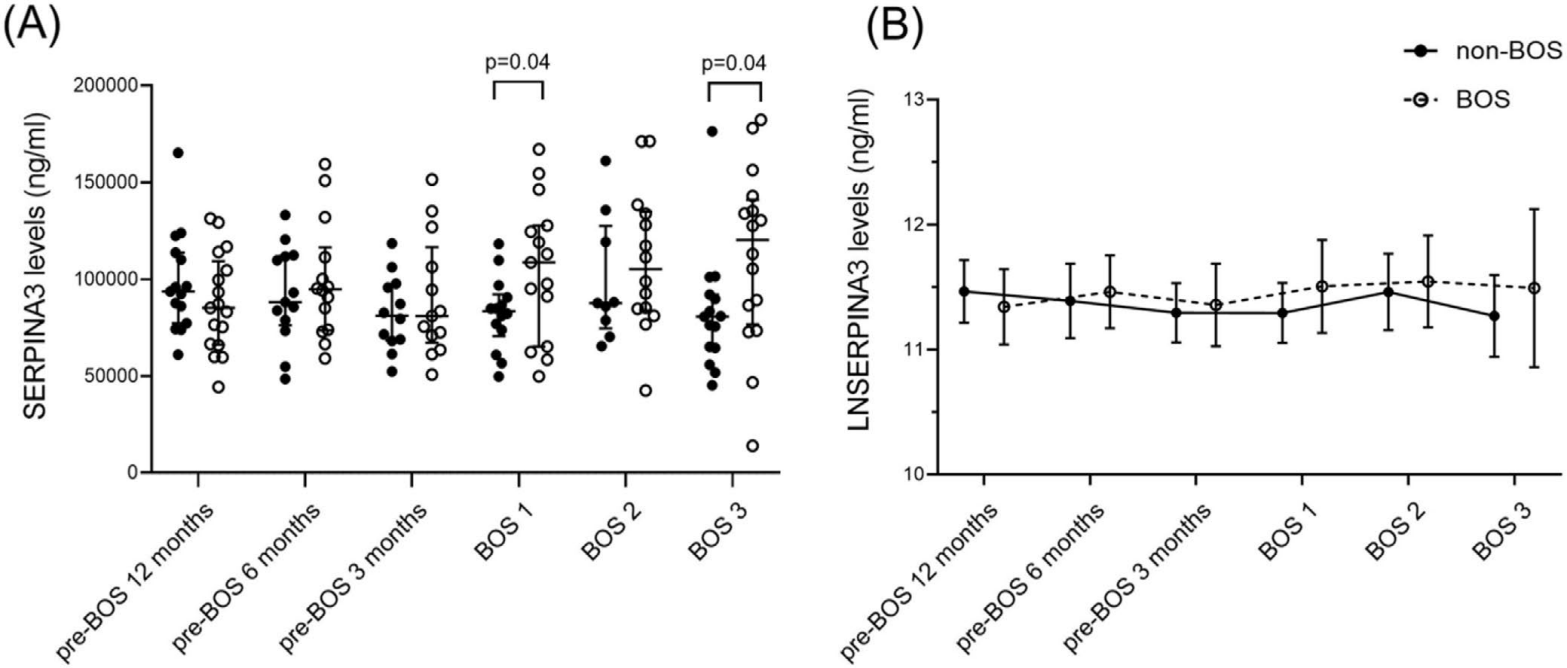
Serum SERPINA3 levels in patients with and without BOS. Panel A: Serum samples from patients who did (*n* = 19) or did not (*n* = 19) develop BOS, from different time points relative to BOS diagnosis in the affected patients, as analyzed by ELISA. All points represent, at each timepoint, measurements from individual lung transplantation patients. Error bars reflect median [interquartile range]. Groups were compared at each time point using a Mann–Whitney *U*; *p* < 0.05 was considered significant. BOS, bronchiolitis obliterans syndrome. Panel B: Longitudinal mean ± standard deviation of LN transformed serum SERPINA3 levels in ng/ml in non‐BOS patients and BOS patients over different timepoints. The solid line represents the non‐BOS group; the dotted line represents the BOS group. Error bars indicate the standard deviation. BOS, bronchiolitis obliterans syndrome.

SERPINA3 levels in serum were significantly higher in patients with BOS compared to non‐BOS patients at BOS stage 1 and BOS stage 3 (Figure 3A). SERPINA3 levels in serum correlated negatively to FEV1 in liters (estimate −0.421, 95% CI −0.695 to −0.135, *p* < 0.005). In the 12 months before onset of BOS (12 months before BOS until BOS stage 1) LnSERPINA3 levels decreased in non‐BOS patients per day before BOS onset, while LnSERPINA3 increased in BOS patients per day before BOS onset (−0.015 (95% CI −0.29 to −0.001) vs. 0.01 (95% CI 0.005–0.044), *p* = 0.01) (Figure 3B).

### 3.2 Low SERPINA3 Deposition in Partially and Completely Obstructed Airways Colocalizes With Mast Cells in the Lung Tissue From Patients With BOS

**FIGURE 4:**
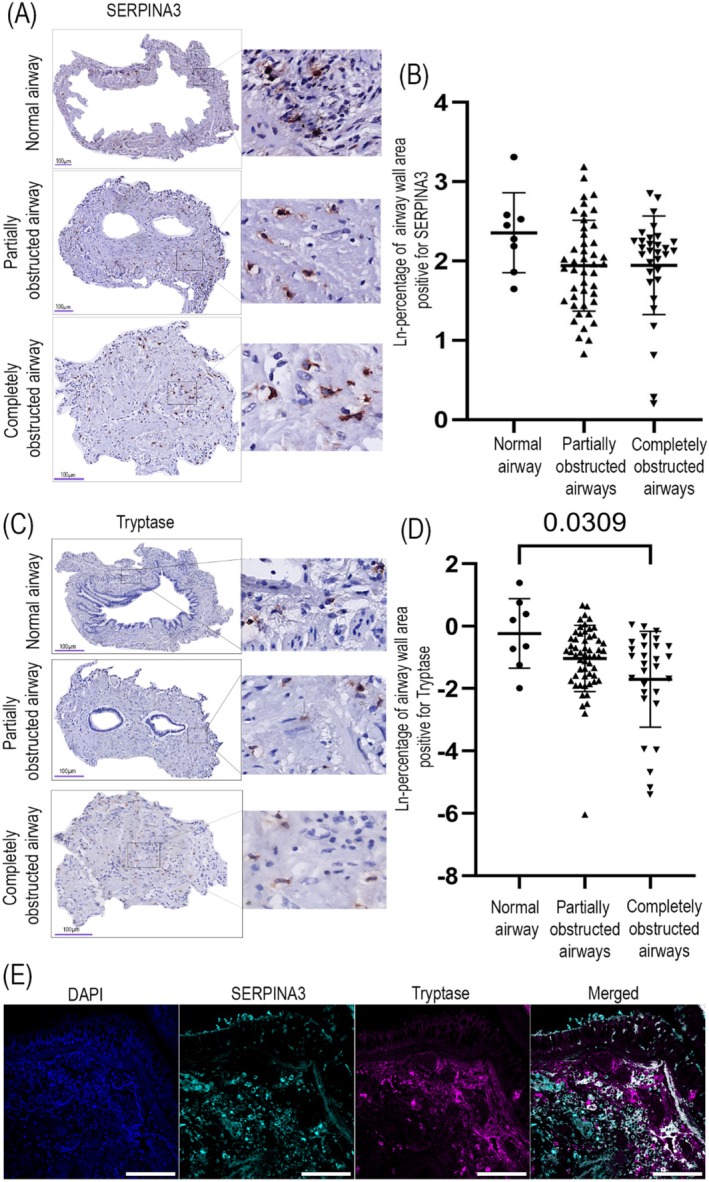
Localization of SERPINA3 and tryptase‐positive mast cells in airways in BOS lung tissue. Formalin‐fixed paraffin‐embedded lung tissue from patients with BOS stained for SERPINA3 using immunohistochemistry. (A) SERPINA3‐positive signal is shown in red as detected by Nova Red; nuclei are shown in blue as detected with hematoxylin. Sections were imaged at original objective magnification 400×, and digital magnified images are shown next to each representative image. (B) The percentage of total tissue area positive for SERPINA3 in normal, partially and completely obstructed airways is presented as ln‐transformed values and each datapoint represents an individual airway. Data are presented as mean ± SD and groups were compared using a linear mixed effects regression analysis (*n* = 6 patients, number of airways per patient: 0–6 for normal airways, 0–21 for partially obstructed airways, 0–23 for completely obstructed airways). (C) Formalin‐fixed paraffin‐embedded lung tissue from patients with BOS stained for tryptase using immunohistochemistry. Tryptase‐positive mast cells localized to normal airways, partially and completely obstructed airways. (D) The percentage of total tissue area positive for tryptase in normal, partially and completely obstructed airways and is presented as ln‐transformed values with each datapoint representing an individual airway (*n* = 6 patients, number of airways per patient: 0–6 for normal airways, 0–21 for partially obstructed airways, 0–19 for completely obstructed airways). Data are presented as mean ± SD and groups were compared using linear mixed effects regression analysis, *p* < 0.05 was considered statistically significant. (E) Colocalization of SERPINA3 and tryptase in lung tissue from patients with BOS stained for SERPINA3 and tryptase using immunofluorescence. Cell nuclei, SERPINA3, and tryptase are respectively shown in blue, cyan, and purple, colocalized regions are represented in white in the merged image (scale bar = 100 μm).

The elevated serum SERPINA3 levels in patients with BOS prompted us to further investigate the localization of SERPINA3 in lung tissues from patients with BOS. Therefore, we used immunohistochemistry to identify SERPINA3. As it was not possible to access lung tissue samples from lung transplant patients without BOS to use as controls, we used SERPINA3 distribution in unaffected/normal airways in lung tissue from patients with BOS to compare to partially and completely obstructed airways in the same tissues (Figure 4A). We observed that in normal airways SERPINA3 localized in the adventitia and was specifically detected in a population of cells with a distinctive round morphology. In partially obstructed airways, the cells positively stained for SERPINA3 were mainly observed around the smooth muscle area and partly in the obstructed connective tissue area of the airway. When airways were completely obstructed, the SERPINA3 staining was more prominently present in the connective tissue in the luminal obstruction (Figure 4A). Subsequently, we quantified the percentage area of tissue with positive SERPINA3 staining within the whole airway wall excluding epithelium. The ln‐transformed data indicated a lower percentage area positive for SERPINA3 in both partially and completely obstructed airways than in normal airways, which is likely mainly the result of the increase in connective tissue present in the scarred obstructed area (Figure 4B).

The cells positively stained for SERPINA3 were number‐ and distribution‐wise suggested to be mast cells. To confirm this supposition, we stained for tryptase, which is only expressed by mast cells. In lung tissue sections from patients with BOS, we observed tryptase expression in all categories of airways with a similar distribution as SERPINA3 (Figure 4C). Next, we calculated the percentage of staining area for tryptase‐positive mast cells within each entire airway. We found that tryptase‐positive mast cell area was significantly lower in obstructed airways compared to normal airways (Figure 4D). To explore whether SERPINA3‐stained cells were mast cells, colocalization between SERPINA3 and tryptase‐positive mast cells was determined by immunofluorescence in the airways of BOS lung tissue. We observed SERPINA3 and tryptase colocalized, indicating the SERPINA3‐positive cells were probably tryptase‐positive mast cells in the small airways from transplant patients with BOS (Figure 4E).

### 3.3 More Chymase‐Positive Mast Cells in Partially Obstructed Airways in BOS Lung Tissue

**FIGURE 5:**
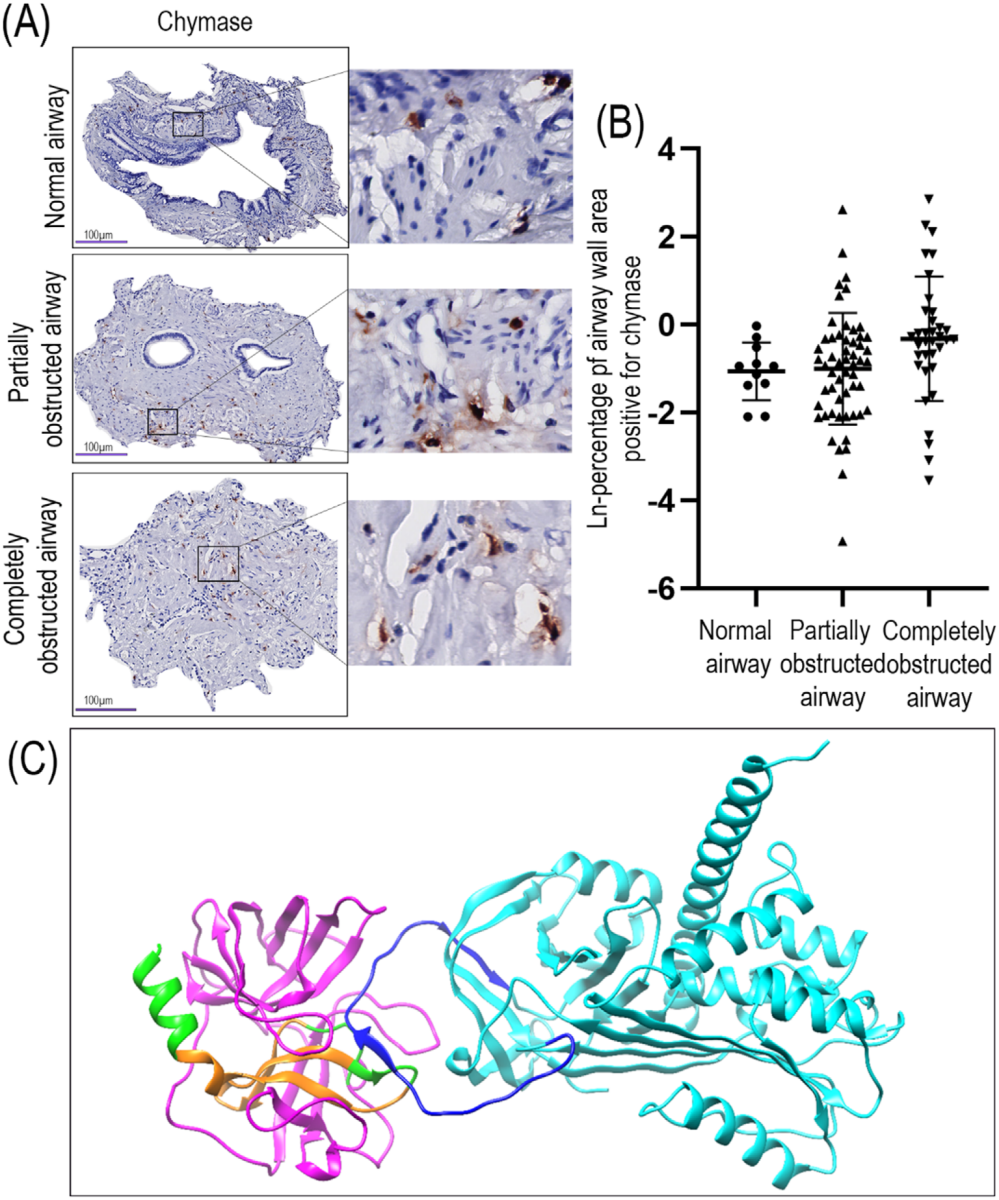
Localization of chymase‐positive mast cells in airways of BOS lung tissue. Airways in lung tissue from patients with BOS stained for chymase using immunohistochemistry. Chymase expression is shown in red detected with NovaRed; nuclei are shown in blue detected with hematoxylin. Sections were imaged at the original objective magnification 400× and digital magnified images are shown next to each representative image. Three categories of airways were identified; normal, partially and completely obstructed airways. (A) Chymase‐positive mast cells localized to normal airway, partially obstructed airway, and completely obstructed airway. (B) Percentages of chymase‐positive area within normal, partially obstructed, and completely obstructed airways are presented as ln‐transformed values and each datapoint represents an individual airway. Differences were analyzed using linear mixed effects regression. Data are presented as mean ± SD, *p* < 0.05 was considered statistically significant (*n* = 6 patients, number of airways per patient: 0–9 for normal airways, 0–21 for partially obstructed airways, 0–23 for completely obstructed airways). (C) AlphaFold model showing the possible interaction of SERPINA3 with chymase and the location of the chymase antibody binding site. SERPINA3 is shown in cyan with the reactive center loop of the molecule (amino acids (AA) 369–394) shown in blue. Chymase is represented in magenta, with the immunogenic peptide (AA 198–247) region shown in green, and the predicted epitopes for interaction with the antibody shown in orange (AA 204–219 and 224–237).

Chymase, a serine protease released from mast cells, can be inhibited by SERPINA3 through the formation of a stable complex which impedes the enzymatic function of chymase [10]. Given this interaction, we were interested in the relationship between chymase and SERPINA3 expression by mast cells in the different airways in BOS lung tissue. We detected chymase‐positive mast cells in connective tissue in normal airways (Figure 5A), whereas in partially obstructed airways we detected them in the connective tissue surrounding airway smooth muscle. In completely obstructed airways, these cells distributed to the connective tissue in the luminal obstruction. In fully obstructed airways we found a trend toward a higher area of chymase positivity compared to normal and partially obstructed airways (Figure 5B). To further investigate the relationship between chymase and SERPINA3, we investigated the extent of overlap between staining for chymase and SERPINA3. Surprisingly, we found only limited colocalization between the positive signals for chymase and SERPINA3 (Figure S2). We then questioned whether the binding between chymase and SERPINA3 was interfering with the detection by the anti‐chymase antibody we applied. We therefore investigated potential protein–protein interactions, modeling the binding relationship between SERPINA3 and chymase, and identifying the location of the chymase antibody binding site (Figure 5C). This prediction indeed indicated that SERPINA3 and chymase would strongly interact at a site that directly overlaps with the antibody binding site (Figure 5C). This is indicated by the alignment of the blue reactive center loop in the SERPINA3 molecule and the chymase antibody binding region with the chymase molecule shown in green and orange in Figure 5C. This modeling therefore suggested that our chymase antibody could not detect chymase when it was bound with SERPINA3. Consequently, the chymase that we did observe in airways was then not bound to SERPINA3 (and is referred to as unbound chymase from here on), indicating that we observed greater levels of unbound chymase in fully obstructed airways in BOS.

### 3.4 Lower OPG Deposition in Partially and Completely Obstructed Airways, Which Colocalized With SERPINA3 and Tryptase in Lung Tissue From Patients With BOS

**FIGURE 6:**
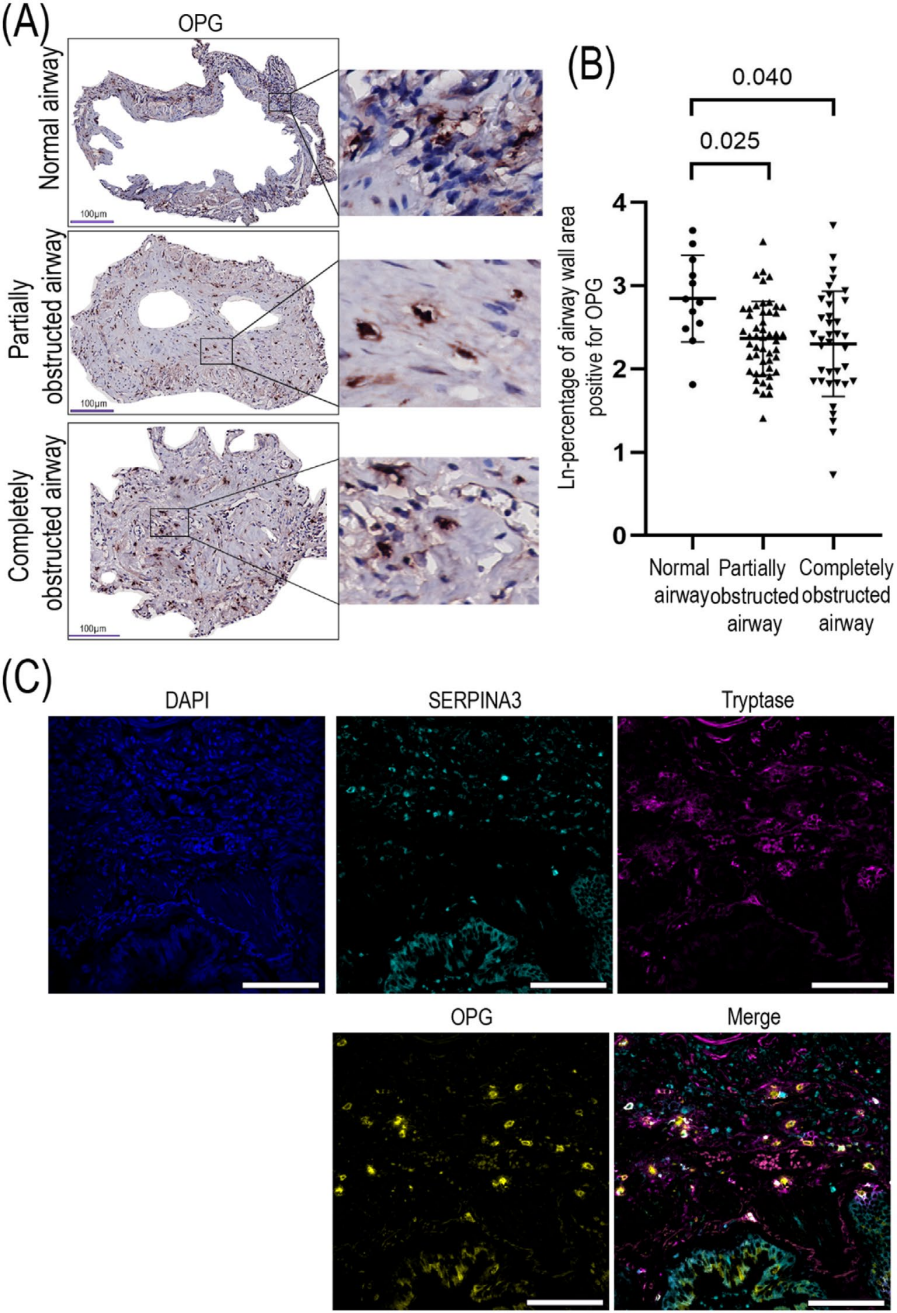
Localization of OPG in airway walls. Airways in lung tissue from patients with BOS were stained for OPG using immunohistochemistry. (A) OPG expression is shown in red detected with Nova Red; nuclei are shown in blue detected with hematoxylin. Sections were imaged at original objective magnification 400×, and digital magnified images are shown next to each representative image. (B) The percentage of OPG‐positive area in normal, partially and completely obstructed airways is presented as ln‐transformed values, and each datapoint represents an individual airway. Differences were analyzed using linear mixed effects regression. Data presented as mean ± SD, *p* < 0.05 was considered statistically significant (*n* = 6 patients, number of airways per patient: 0–10 for normal airways, 0–23 for partially obstructed airways, 0–26 for completely obstructed airways). (C) The colocalization of SERPINA3, tryptase and OPG in lung tissue from patients with BOS using immunofluorescence. Cell nuclei, SERPINA3, and tryptase are respectively shown in blue, cyan, and purple, colocalized expression is represented in white in the merged image (scale bar = 100 μm).

Tryptase‐positive mast cells can produce OPG protein [20], a fibrosis associated factor. Moreover, chymase released from mast cells and OPG can negatively regulate each other under certain conditions [31]. We therefore wanted to investigate the expression of OPG in airways of lung tissue from patients with BOS. Immunohistochemistry was again used to locate OPG in the airways of those patients. OPG expression (Figure 6A) had a similar distribution pattern as seen for SERPINA3 and tryptase. We observed more OPG in normal airways compared to partially and completely obstructed airways (Figure 6B). Subsequently, we investigated if SERPINA3, tryptase, and OPG colocalized in lung tissue from lung recipients with BOS. Using immunofluorescence, we found cells stained for SERPINA3, tryptase, and OPG localized to the connective tissue of the airways (Figure 6C).

### 3.5 SERPINA3 and OPG May Form a Complex in the Airways of Lung Tissue From Patients With BOS

We were initially interested in whether SERPINA3, chymase, and OPG interacted in obstructed airways of patients with BOS. However, our modeling results showed that chymase was unlikely to be detected with our antibody when bound to SERPINA3. Consequently, we shifted our focus to explore the possibility of interactions between SERPINA3, tryptase (as an indicator of mast cells), and OPG.

**FIGURE 7:**
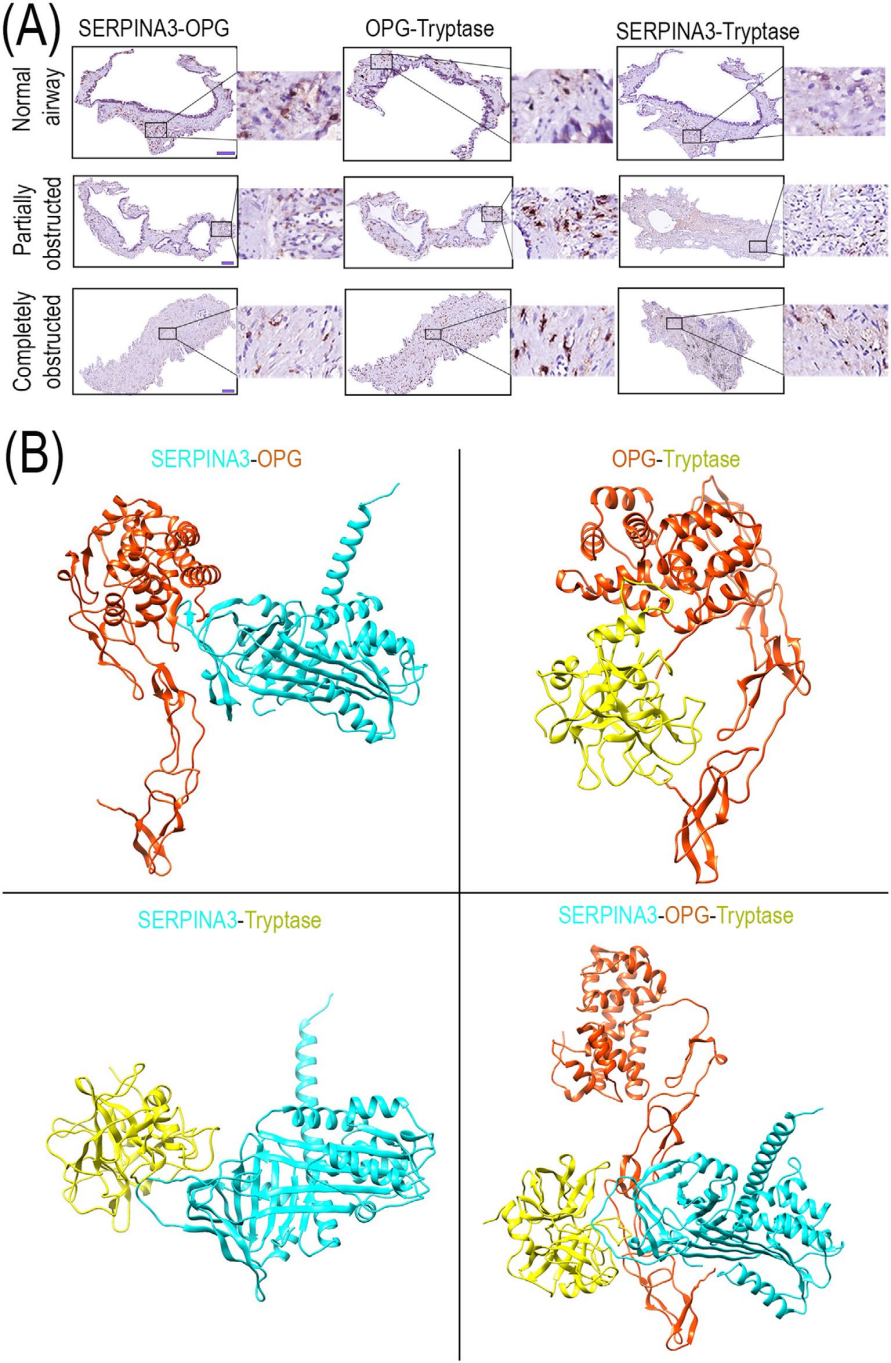
Proximity of SERPINA3, OPG, and tryptase in airways of lung tissue from recipients with BOS. (A) Representative images of proximity detection of SERPINA3 and OPG, OPG and tryptase, SERPINA3 and tryptase in lung tissue obtained from lung recipients with BOS. Each combination of two primary antibodies of SERPINA3, OPG and tryptase were simultaneously incubated with lung tissue. Positive signal (in red) is only found if distance between two proteins is within 40 nm [32]. Sections were imaged at original objective magnification 400× (bar = 100 μm) and digital magnified images are shown next to each representative image. (B) AlphaFold models of possible interactions between SERPIN3A, OPG and tryptase. SERPINA3, OPG and tryptase are shown respectively in cyan, orange and yellow.

A proximity ligation assay was used to investigate whether SERPINA3, tryptase, and OPG were located close enough within airway tissues to interact (Figure 7A). A strong proximity signal was detected between SERPINA3 and OPG, and this signal associated with cells located within connective tissue of all airways. A more diffuse proximity signal was also observed in the extracellular matrix around cells in the connective tissue of partially obstructed airways and within luminal obstructions of completely obstructed airways. Similarly, a strong proximity signal between OPG and tryptase was detected, predominantly associated with cells within the connective tissue of both partially and completely obstructed airways. For SERPINA3 and tryptase, the proximity signal was weaker but displayed a distribution pattern similar to that of OPG and tryptase, suggesting that SERPINA3 and tryptase are potentially in close proximity.

Next, we performed AlphaFold modeling again to predict potential interactions between SERPINA3 and OPG, OPG and tryptase, SERPINA3 and tryptase, as well as OPG, SERPINA3, and tryptase together (Figure 7B). This modeling showed the potential for a strong interaction between SERPINA3 and OPG, a moderate interaction between OPG and tryptase, a weak interaction between SERPINA3 and tryptase, and a hypothetical interaction between SERPINA3, OPG, and tryptase as inferred from the stabilizing energy of the different interactions (Table 8).

**TABLE 8:** Analysis of biomolecular interactions.

Alpha fold run id	Proteins	Pdb/Uniprot	ipTM	pTM	H bond kJ/mol	Electro static kJ/mol	Van der Walls kJ/mol	Total stabilizing kJ/mol
Test 1klt 3dlw 2024‐07‐12_18:37	Chymase SERPINA3	1KLT 3DLW	0.8	0.63	−122.85	15.81	−369.94	−476.98
Serpina3_chymase	SERPINA3 Chymase	P01011 (24–423) P23946 (22–247)	0.79	0.64	−110.99	−7.42	−316.79	−435.2
2024‐08‐20_10:00	SERPINA3 Chymase	P01011 (1–423) P23946 (1–247)	0.76	0.64	−104.31	−17.49	−321.57	−443.37
Serpina3_OPG	SERPINA3 OPG	P01011 (24–423) O00300 (22–401)	0.17	0.5	−71.79	−72.36	−220	−364.15
2024‐10‐06_20:55	SERPINA3 OPG	P01011 (1–423) O00300 (1–401)	0.16	0.48	−56.95	−19.57	149.87	73.35
OPG_Tryptase	OPG Tryptase	O00300 (22–401) Q15661 (31–275)	0.18	0.47	−28.88	0.17	−247.63	−276.35
2024‐10‐14_19:55	OPG Tryptase	O00300 (1–401) Q15661 (1–275)	0.51	0.55	−27.81	−73.14	−170.03	−270.98
SERPINA3_Tryptase	SERPINA3 Tryptase	P01011 (24–423) Q15661 (31–275)	0.42	0.61	−92.2	−9.06	2.31	−98.95
2024‐10‐14_19:54	SERPINA3 Tryptase	P01011 (1–423) Q15661 (1–275)	0.42	0.57	−112.47	−91.28	−287.59	−491.34
SERPINA3_Tryptase_OPG	Tryptase (a) SERPINA3 (b) OPG (c)	Q15661 (31–275) P01011 (24–423) O00300 (22–401)	0.3	0.46	−88.91 −7.44 −18.91	−115.82 −11.52 −38.78	−322.8 −66.55 −31.49	−527.53 a/b −85.51 a/c −89.18 b/c
2024‐10‐14_19:52	SERPINA3 (a) OPG (b) Tryptase (c)	P01011 (1–423) O00300 (1–401) Q15661 (1–275) O00300 (1–401)	0.27	0.44	−184.76 −124.59 0	−93.94 −91.73 −5.86	2263.94 −365.10 −12.02	1985.24 a/b −581.42 a/c −17.87 b/c

Altogether these data suggest that SERPINA3 and OPG may be released from mast cells and possibly exhibit an interaction outside cells in the connective tissue of airways of BOS lung tissue.

### 3.6 SERPINA3 and OPG Are Positively Correlated in Partially and Completely Obstructed Airways in Lung Tissue From Patients With BOS

**FIGURE 8:**
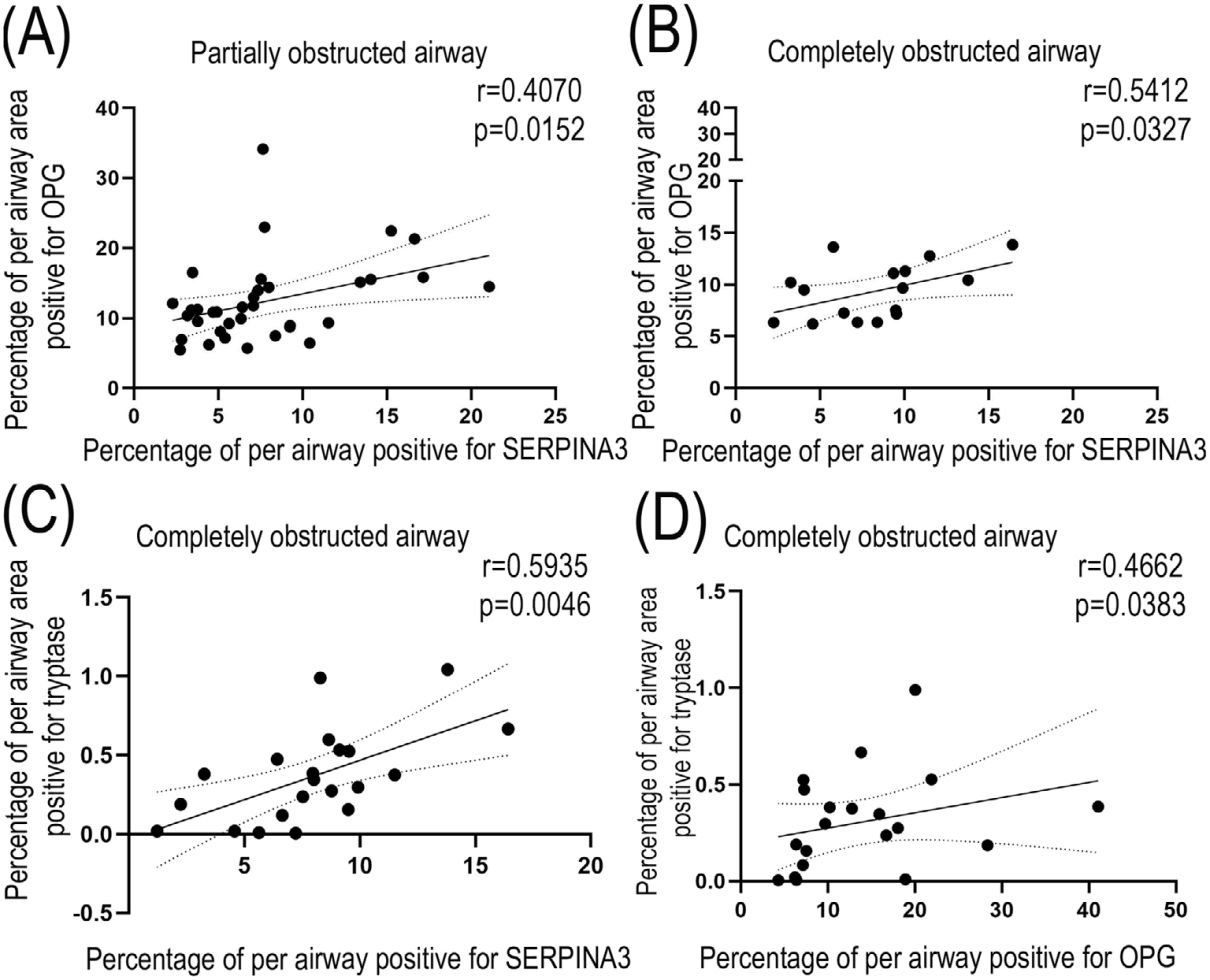
Correlations between expression of OPG and SERPINA3 or OPG and tryptase in partially and completely obstructed airways in lung tissue from recipients with BOS. (A) Correlation between SERPINA3 and OPG in partially obstructed airways (*n* = 35 airways). (B) Correlation between SERPINA3 and OPG in completely obstructed airways (*n* = 16 airways). (C) Correlation between SERPINA3 and tryptase in completely obstructed airways (*n* = 21 airways). (D) Correlation between OPG and tryptase in completely obstructed airways (*n* = 20 airways). Correlations were tested using a Spearman test, *p* < 0.05 was considered significant.

Since a potentially strong interaction between SERPINA3 and OPG was observed, we investigated whether their expression correlated in obliterated airways of patients with BOS. In normal airways, we found a correlation coefficient value between SERPINA3 and OPG of 0.77, which was not significant (Figure S3), which may have been caused by the small sample size of normal airways in our cohort. In partially and completely obstructed airways (Figure 8A,B), however, SERPINA3 and OPG expression exhibited a positive correlation. Recognizing SERPINA3 and OPG may be produced by tryptase‐positive mast cells, we also examined the correlation between tryptase and SERPINA3, as well as tryptase and OPG. The data revealed no correlation between tryptase and SERPINA3 or tryptase and OPG in normal or partially obstructed airways (Figure S4). This is probably also due to the lack of power caused by the small sample size. In completely obstructed airways, however, we found a positive correlation between tryptase and SERPINA3, as well as between tryptase and OPG, in airways from patients with BOS. These data support our finding that OPG and SERPINA3 may be released from tryptase‐positive cells and deposited in the connective tissue of airway walls in lung tissue from patients with BOS.

## 4 Discussion

In this study, we investigated the presence of SERPINA3 and OPG and their potential interaction in airways of lung tissue from patients with BOS after lung transplantation. We found similar distribution patterns of SERPINA3 and OPG, and we detected tryptase‐positive mast cells in the submucosa of airways. Presence of these cells coincided with the cells positively stained for SERPINA3 and OPG, indicating SERPINA3 and OPG are possibly produced by tryptase‐positive mast cells in BOS. Furthermore, we found that these two proteins were also close together in the extracellular space, suggesting tryptase‐positive mast cells may deposit them in the extracellular microenvironment. Overall, our findings suggest that SERPINA3 and OPG are likely released from tryptase‐positive mast cells, and the association between the expression of SERPINA3 and OPG may be involved in airway obstruction in BOS.

The lower percentage SERPINA3 and OPG expression in partially and completely obstructed airways was likely a consequence of the increased amount of extracellular matrix present in the connective tissue and luminal obstruction in these abnormal airways. Similarly, the percentage area of tryptase‐positive mast cells would also be impacted by the increase in extracellular matrix as obstruction of the airways increases. Therefore, the absolute amount of SERPINA3, OPG, and tryptase may not have changed or may even be increased within these regions; however, the main localization of these proteins was different in BOS, with less in the submucosa and more in the obliterating connective tissue in the region that was the lumen in obstructed airways. This differential localization suggests a role in the pathological process of BOS.

BOS has been recognized as a complex lung pathology with excessive extracellular matrix accumulation contributing to airway obstruction. Considering that our data suggest that SERPINA3 and OPG may be produced by tryptase‐positive mast cells, which we found present around BOS airways, it is plausible to speculate that these two proteins are mediators playing a role in the process of mast cell‐driven airway obstruction. During BOS, when the airways are partially obstructed, the resident tryptase‐positive mast cells may migrate to the submucosa and connective tissue of the injured airways. Subsequently, SERPINA3 and OPG could be released from these cells. At advanced stages of airway obstruction, with high presence of SERPINA3 and OPG, these proteins may then diffuse to the connective tissue in the luminal obstruction. When SERPINA3 and OPG are released from tryptase‐positive mast cells into the extracellular environment, these proteins may interact with fibrosis‐associated proteins to regulate extracellular matrix remodeling. Clinically, inhibition of mast cells by for example montelukast (a leukotriene antagonist) started directly after lung transplantation could ameliorate the role of these mast cells, as well as SERPINA3 and OPG. A small prospective study performed with montelukast in progressive BOS patients [33] did show attenuation of FEV1 decline but only for patients with BOS stage 1. The moment of starting montelukast could have influenced results, since irreversible fibrotic changes were already present in the later stages of BOS.

SERPINA3 has emerged as a novel fibrotic marker associated with various fibrotic disorders [34, 35]. It plays a role as an essential protein in regulation of extracellular matrix gene expression in certain conditions. Matthew et al. [14] reported that silencing SERPINA3 resulted in low gene expression of collagen II, collagen IX and collagen XI in mesenchymal stem cells characterized by chondrogenesis. OPG is a glycoprotein shown to be involved in regulating extracellular matrix remodeling, for instance through its affinity for matrix proteins such as fibulin‐1 [36] and connective tissue growth factor (CTGF) [37]. Both fibulin‐1 [38] and CTGF [39] have been recognized as important regulators of fibrotic processes. This knowledge implies that OPG may play roles in regulating extracellular matrix deposition, resulting in the airway obstruction in BOS. Given the proximity and the association we observed between SERPINA3 and OPG in affected airways, we speculate that these two proteins may exhibit a synergistic relationship with each other in regulating obstruction of airways. An open question remains that needs further consideration: Do SERPINA3 and OPGprobablyreleased from tryptase‐positive mast cells, act as mediators to further stimulate the response of fibroblasts to produce more extracellular matrix in the injured airways of BOS?

An interesting puzzle in our study is the increase in both unbound chymase in lung tissue and SERPINA3 in serum of patients with BOS. The role of chymase is worth considering in the process leading to the development of airways obstruction, even though the chymase that was detected in this study was not in the form that was bound to SERPINA3, but rather the unbound chymase. SERPINA3 is an acute phase protein that is secreted by the liver in response to inflammation [12]. Therefore, the elevated level in serum could be coming from the liver. What the contribution of the lung is to circulating levels of SERPINA3 remains an open question. We noted that SERPINA3 was present in cells and the extracellular space in the submucosa and intraluminal of airways, where the percentage of unbound chymase detected per tissue area was greater in the completely obstructed airways, compared to unobstructed airways. A regulatory link between chymase in obstructed airway tissues and serum SERPINA3 in BOS remains elusive. We speculate that a feedforward signal provided by an increase in unbound chymase resulting from, for example, infection preceding BOS could enhance liver production of acute phase proteins including SERPINA3. In other cells, chymase has been shown to negatively regulate OPG: when human osteoblasts were stimulated with chymase, OPG protein was reduced [31]. This potentially suggests that the high levels of unbound chymase in the obstructed airway may contribute to the downregulation of OPG expression in tryptase‐positive mast cells. However, as the decreased proportion of OPG in completely obstructed airways was predominantly due to the increase in extracellular matrix driving an increase in tissue area, rather than a change in the apparent absolute amount of OPG detected in these airways, this seems unlikely. Further studies are needed to elucidate the impact of chymase on OPG in airway obstruction in BOS.

A limitation in this study is the lack of a causality link between our results and the development or progression of BOS. We have identified new potential molecular interactions in BOS using immunohistochemical staining of BOS lung tissue, proximity assays, and AlphaFold modeling. However, further research is needed to mechanistically validate these potential interactions in BOS and answer remaining questions regarding the involvement of these markers in BOS. Another limitation is the unavailability of control explant tissue from lung transplantation patients without BOS. While all transplant patients undergo routine screening post transplantation, including surveillance biopsies, due to the patchy appearance of BOS and the size of surveillance biopsies from allografts, small airway analysis, as described in this study, cannot be performed on such biopsies. In this study, the unaffected airways in the explant tissue of BOS patients served as control; however, for future studies, access to control explant tissue from LTx patients is preferred. Also, updated criteria by ISHLT (International Society for Heart & Lung Transplantation) for BOS staging have been implemented. However, due to the retrospective nature of this study, guidelines available at the time of selection of serum samples were maintained for the duration of the study.

In conclusion, this study indicated SERPINA3 and OPG are likely produced by tryptase‐positive mast cells, and they potentially form an extracellular complex in BOS airways with a possible role in the submucosal/intraluminal fibrosis. Further investigation is required to elucidate the precise mechanisms underlying obstruction of airways in BOS. An interesting focus could be how OPG and SERPINA3 influence extracellular matrix deposition. By identifying the potential interaction of OPG and SERPINA3, this study highlights a possible novel target for addressing the airway obstruction of fibrotic lesions in BOS lung disease.

## Author Contributions

Yanzhe Liu and Eline A. van der Ploeg contributed equally to this work, were responsible for the design and execution of experiments, data analyses and interpretation, and drafting of the manuscript. Theo Borghuis and R. Ian Menz assisted with execution of experiments, data analyses, interpretation, and manuscript revision. Wim Timens and Judith M. Vonk provided critical advice on experimental design, data interpretation, and manuscript revision. Barbro N. Melgert, C. Tji Gan, and Janette K. Burgess conceived and supervised the study, revised the manuscript critically for important intellectual content, ensured that questions related to the accuracy or integrity of any part of the work are appropriately investigated and resolved, and they are co‐corresponding authors. All authors approved the final version of the manuscript and agree to be accountable for all aspects of the work.

## Funding

Janette K. Burgess acknowledges support from the Nederlandse Organisatie voor Wetenschappelijk Onderzoek (NOW) (Aspasia 015.013.010). C. Tji Gan received a grant from Noordelijke Cara Stichting for this research. Yanzhe Liu is supported by a scholarship provided by the Chinese Scholarship Council and supported by the Graduate School of Medical Sciences of the University Medical Centre Groningen.

## Conflicts of Interest

W.T. reports incidental consultancy fees from Merck Sharp Dohme, Bristol‐Myers‐Squibb, and Altana (all paid to Institution) and roles as a member of the Council for Research and Innovation of the Federation of Medical Specialists and the Program Committee post‐covid ZonMW (unpaid). J.K.B. reports research funds received from Boehringer Ingelheim (not related to this work and paid to Institution) and roles as a board member of the American Thoracic Society (May 2022–May 2024), Netherlands Respiratory Society, and Netherlands Matrix Biology Society (unpaid). All other authors declare they have no conflicts of interest associated with this work.

## Supporting information


**Figure S1:** Formalin‐fixed paraffin‐embedded lung tissue from patients with BOS stained for SERPINA3, OPG and rabbit IgG isotype control using immunohistochemistry. The positive signals are shown in red as detected by Nova Red; nuclei are shown in blue as detected with hematoxylin. Sections were imaged at original objective magnification 400× (bar = 100 μm).

**Figure S2:** Colocalization of SERPINA3 and chymase in lung tissue from patients with BOS. FFPE lung tissue sections from patients with BOS stained with SERPINA3 and chymase using immunofluorescence. SERPINA3, and chymase are respectively shown in yellow, and purple (scare bar = 100 μm).

**Figure S3:** Correlation between SERPINA3 and OPG in normal airways in lung tissue from patients with BOS (*n* = 6 airways, *r* = 0.7714, *p* = 0.1028). Correlations were calculated using a Spearman test.

**Figure S4:** Correlation between tryptase and SERPINA3, tryptase and OPG in normal airways and partially obstructed airways. (A) Correlation between tryptase and SERPINA3 in normal airway (*n* = 5, *r* = 0.000, *p* > 0.999), (B) Correlation between tryptase and OPG in normal airway (*n* = 4, *r* = 0.400, *p* = 0.7500), (C) Correlation between tryptase and SERPINA3 in partially obstructed airway (*n* = 32, *r* = 0.2093, *p* = 0.2503), (D) Correlation between tryptase and SERPINA3 in partially obstructed airway (*n* = 35, *r* = −0.04062, *p* = 0.8168). Correlations were calculated using a Spearman test, *r* = 0.7714, *p* = 0.1028.

## Data Availability

All data generated or analyzed during this study are included in this article and its Supporting Information files. Further enquiries can be directed to the corresponding author.
