# Supplementary figures and images for: A Potential Novel Molecular Interaction in Bronchiolitis Obliterans Syndrome in Lung Transplantation Patients: The Role of SERPINA3 and Osteoprotegerin
Source: FASEB J. 2026 Apr 20;40:e71748. doi: 10.1096/fj.202503694R (PMC13094457; doi:10.1096/fj.202503694R)

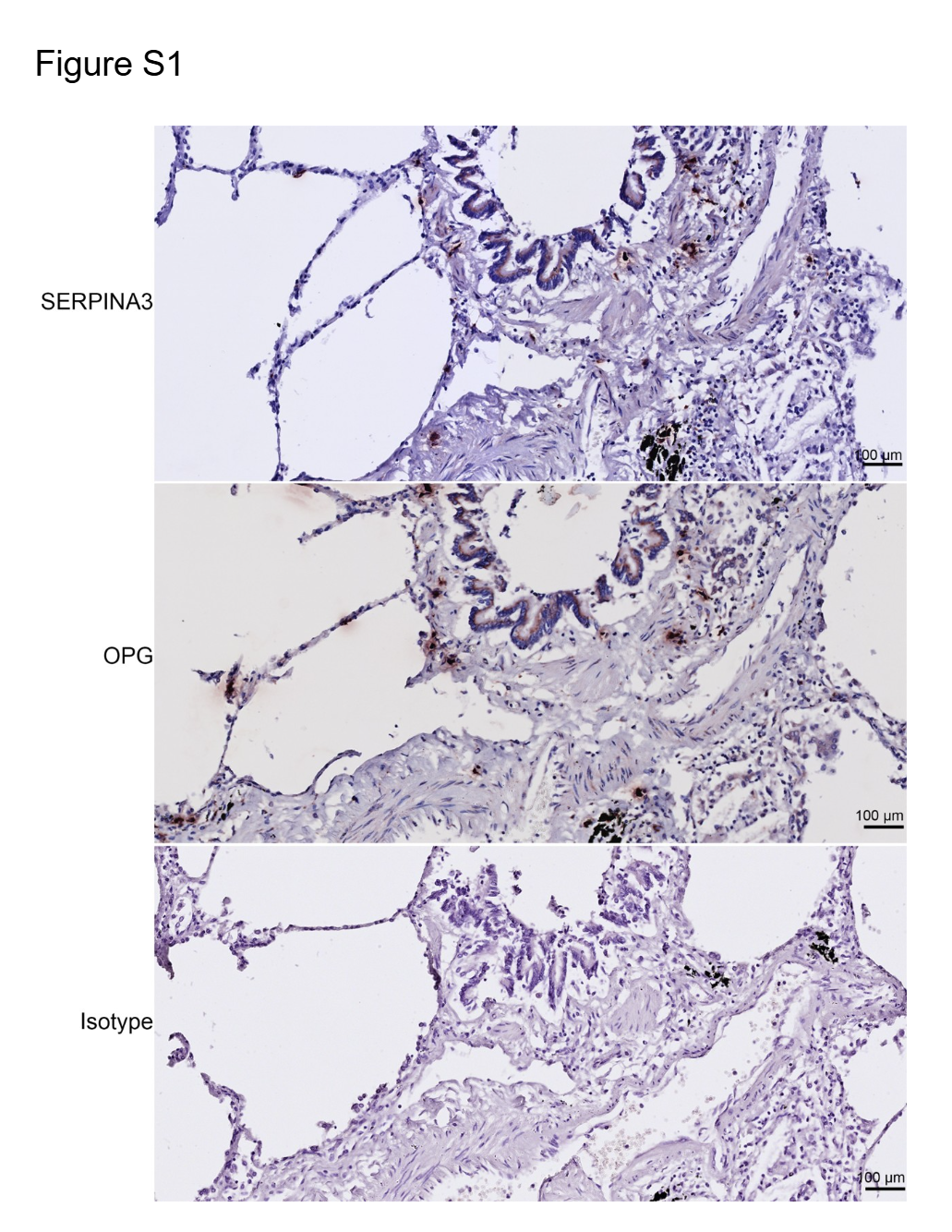

Supplement: Supplementary file 1 — Figure S1: Formalin‐fixed paraffin‐embedded lung tissue from patients with BOS stained for SERPINA3, OPG and rabbit IgG isotype control using immunohistochemistry. The positive signals are shown in red as detected by Nova Red; nuclei are shown in blue as detected with hematoxylin. Sections were imaged at original objective magnification 400× (bar = 100 μm). [file FSB2-40-e71748-s003.png]

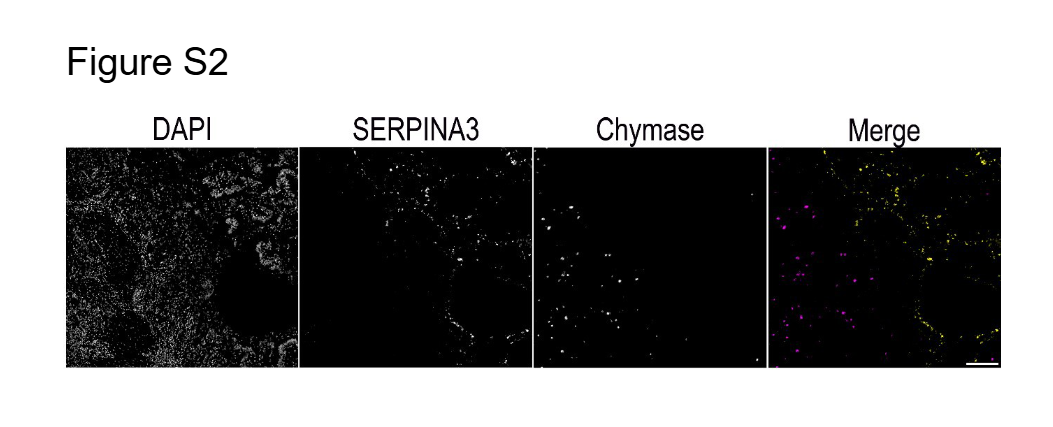

Supplement: Supplementary file 2 — Figure S2: Colocalization of SERPINA3 and chymase in lung tissue from patients with BOS. FFPE lung tissue sections from patients with BOS stained with SERPINA3 and chymase using immunofluorescence. SERPINA3, and chymase are respectively shown in yellow, and purple (scare bar = 100 μm). [file FSB2-40-e71748-s004.png]

Figure S3

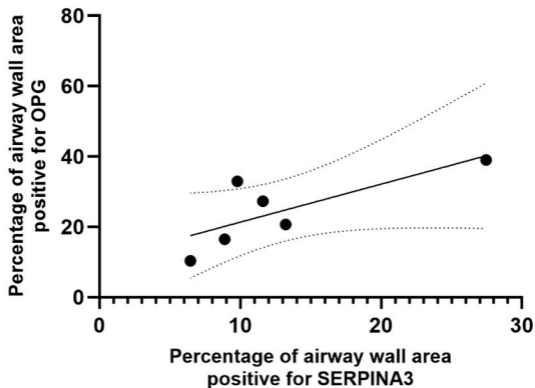

Supplement: Supplementary file 3 — Figure S3: Correlation between SERPINA3 and OPG in normal airways in lung tissue from patients with BOS (n = 6 airways, r = 0.7714, p = 0.1028). Correlations were calculated using a Spearman test. [file FSB2-40-e71748-s002.pdf]

Figure S4

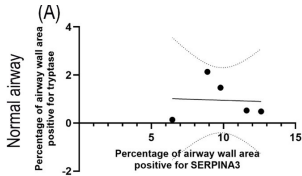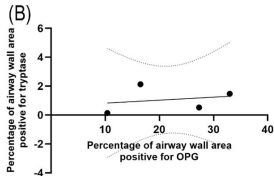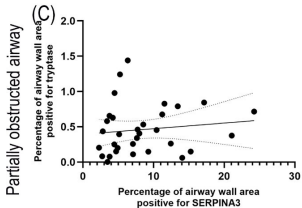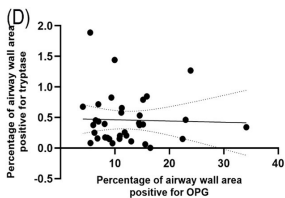

Supplement: Supplementary file 4 — Figure S4: Correlation between tryptase and SERPINA3, tryptase and OPG in normal airways and partially obstructed airways. (A) Correlation between tryptase and SERPINA3 in normal airway (n = 5, r = 0.000, p > 0.999), (B) Correlation between tryptase and OPG in normal airway (n = 4, r = 0.400, p = 0.7500), (C) Correlation between tryptase and SERPINA3 in partially obstructed airway (n = 32, r = 0.2093, p = 0.2503), (D) Correlation between tryptase and SERPINA3 in partially obstructed airway (n = 35, r = −0.04062, p = 0.8168). Correlations were calculated using a Spearman test, r = 0.7714, p = 0.1028. [file FSB2-40-e71748-s001.pdf]
